# Electromagnetic Short-Term to Imminent Forecast Indices for M ≥ 5.5 Earthquakes in the Gansu–Qinghai–Sichuan Region of China

**DOI:** 10.3390/s24123734

**Published:** 2024-06-08

**Authors:** Xia Li, Ye Zhu, Lili Feng, Yingfeng Ji, Weiling Zhu

**Affiliations:** 1 State Key Laboratory of the Tibetan Plateau Earth System, Environment and Resources (TPESER), Institute of Tibetan Plateau Research, Chinese Academy of Sciences, Beijing 100101, China; lxqhdz@163.com (X.L.); zhuye@itpcas.ac.cn (Y.Z.); zhuweiling@itpcas.ac.cn (W.Z.); 2 Qinghai Earthquake Administration, Xining 810001, China; dzj350102@163.com; 3 University of Chinese Academy of Sciences, Beijing 100049, China

**Keywords:** electromagnetic anomaly, earthquake forecast, Gansu–Qinghai–Sichuan area

## Abstract

Electromagnetic indices play a potential role in the forecast of short-term to imminent M ≥ 5.5 earthquakes and have good application prospects. However, despite possible progress in earthquake forecasting, concerns remain because it is difficult to obtain accurate epicenter forecasts based on different forecast indices, and the forecast time span is as large as months in areas with multiple earthquakes. In this study, based on the actual demand for short-term earthquake forecasts in the Gansu–Qinghai–Sichuan region of western China, we refined the construction of earthquake forecast indicators in view of the abundant electromagnetic anomalies before moderate and strong earthquakes. We revealed the advantageous forecast indicators of each method for the three primary earthquake elements (time, epicenter, magnitude) and the spatiotemporal evolution characteristics of the anomalies. The correlations between the magnitude, time, intensity, and electromagnetic anomalies of different M ≥ 5.5 earthquakes indicate that the combination of short-term electromagnetic indices is pivotal in earthquake forecasting.

## 1. Introduction

Earthquakes are natural phenomena that occur in the Earth’s interior and are the result of interactions among the Earth’s interior media. In recent decades, studies of regional seismicity in different regions have proposed a variety of new techniques for seismicity-based earthquake forecasting, providing a new possible impetus for feasible earthquake forecasting in the future (e.g., [1]). In the wake of progress in seismological studies (e.g., [2,3]), meaningful earthquake precursors, including physical parameters such as mechanical deformations, gas emissions, groundwater level variations, ground temperature variations, and electromagnetic signals induced by fluctuations in the electromagnetic field, are becoming a hot topic of earthquake studies (e.g., [4,5,6]). When the rock exceeds its load-bearing pressure, it will crack, and the crystal lattice of the rock will be damaged, thus generating a potential jump, releasing electromagnetic wave signals [7,8], and the ultralow frequency (ULF) signals can propagate from the earthquake source to the Earth surface without strong absorption (e.g., [9,10]). The occurrence of electromagnetic effects in ULF signals prior to an earthquake has been recognized to be statistically correlated with earthquakes (e.g., [11]). For instance, lithospheric electromagnetic emissions in relation to three major earthquakes (M = 6.8~8.5) occurring in the Indian subcontinent include ULF (0.01~10 Hz) and very-low-frequency (3~30 kHz) emissions, and ULF emissions can travel to observation stations via crustal regions prior to mainshocks [12]. The presence of ULF precursory signatures of earthquakes was also identified before the M > 7 earthquakes occurred at Spitak, Loma Prieta, Guam, and Biak (e.g., [13]).

An effective precursor extraction method not only extracts significant abnormal signals but also deepens the understanding of the physical mechanism of earthquake generation (e.g., [14]). In recent years, seismic electromagnetic analysis indices have been used as possible identification clues for seismic electromagnetic anomalies and as a reference basis for earthquake forecasting. Preseismic electromagnetic anomalies have been detected before many M ≥ 5.5 earthquakes, including the 2008 M8.0 Wenchuan earthquake (e.g., [15,16]), 2013 M7.0 Lushan earthquake (e.g., [17,18]), 2017 M7.0 Jiuzhaigou earthquake (e.g., [19]), 2017 M6.9 Milin earthquake (e.g., [20]), 2019 M6.0 Changning earthquake (e.g., [21]), 2021 M7.4 Maduo earthquake (e.g., [22]), and M6.9 Menyuan earthquake (e.g., [23,24]).

However, there remain some controversial problems that deserve further study in the application of current electromagnetic forecast indices. On the one hand, there are many methods of electromagnetic analysis and forecasting, and it is difficult to provide consistent forecast results regarding earthquake epicenters based on multiple forecast indices. For example, a total of eight electromagnetic anomalies were detected before the occurrence of the M6.9 Menyuan earthquake in 2022, but the forecast indicators are inconsistent, and a clear forecast cannot be reached even if an anomaly is extracted before the occurrence of the earthquake. On the other hand, the time span of the forecast is large, from several months to several years, which limits the ability to forecast short-term to imminent earthquakes and cannot play an important role in effective disaster reduction immediately before quakes. For instance, before the occurrence of the 2021 M7.4 Maduo earthquake, electromagnetic anomalies appeared in Qinghai approximately 4 years before the earthquake, and a total of six electromagnetic anomalies were extracted, some of which implied that an earthquake was imminent. Therefore, there are still limitations in the application of multiple electromagnetic analyses and methods for earthquake forecasting. It is of practical significance to carry out further detailed sorting of electromagnetic precursor anomalies in the Gansu–Qinghai–Sichuan area of western China to seek a better systematic index-based forecast method for the three earthquake elements (time, epicenter, magnitude) based on the variability in the detected intensity and frequency of the signals adjacent to the epicenter.

## 2. Methods

Three-component high-resolution fluxgate magnetometers developed by Institute of Geophysics, China Earthquake Administration (Beijing, China) are employed in the China Geomagnetic Observatory Network and provide a large amount of geomagnetic data. The data calculations involve daily second sampling, specific frequency band analysis, and residual analysis and smoothing.

Nine commonly used seismic electromagnetic analysis and forecasting methods have been proposed in recent decades. These methods can be divided into five categories: geoelectric field dominant azimuth methods [17], geomagnetic diurnal-variation anomaly analyses [25,26,27], geomagnetic vertical intensity polarization anomalies [20,28], anomalies of seismic direct current apparent resistivity [29,30,31,32,33], and geomagnetic depth sounding methods of current apparent resistivity [34,35].

The observation environment of seismic electromagnetic fields is becoming increasingly complicated, which makes it more difficult to identify relatively weak signals from station observation data. Physical analysis combining mathematics, signal processing, and seismic electromagnetic physical processes is considered a future development direction, and methods for determining geoelectric field-dominant azimuths are gradually being established. Zhao et al. (2023) [17] analyzed the original geoelectric field data from the Dawu seismic station by using the dominant azimuth method and suggested that the dominant azimuth anomaly based on the electric field has good forecasting efficiency for short- to medium-term earthquakes. Specifically, the geoelectric field dominant azimuth method has a certain anti-interference ability, especially when the multisite dominant azimuth anomaly has a quasisynchronous pattern, and the application of this method has high reliability [17]. Moreover, the calculation process of this method is objective, the calculation results are continuous, and the anomaly interpretation is scientific, which makes it possible to use the geoelectric field to carry out earthquake tracking analysis.

The methods for analyzing geomagnetic diurnal variation anomalies include the geomagnetic low-point displacement method, geomagnetic loading and unloading response ratio method, geomagnetic daily ratio method, geomagnetic diurnal variation spatial correlation method, and geomagnetic daily one-value difference method. These methods can intuitively extract the diurnal aberrations of the geomagnetic vertical component, which may be related to the concentration of crustal-induced current (e.g., [22]). In addition, the geomagnetic daily one-value difference method is developed from the geomagnetic daily one-value spatial correlation method, which overcomes the disadvantage of the correlation reference station affecting anomaly reliability. The forecast index systems of the geomagnetic loading and unloading response ratio method and geomagnetic daily ratio method have greatly improved the accuracy of the forecasts of earthquake magnitude and location [22,36].

The geomagnetic vertical intensity polarization anomaly method was developed based on the results of geomagnetism, in which the amplitude of the vertical component of the magnetic signal from the ionosphere is smaller than that of the horizontal component, and its ratio is less than 1 (e.g., [37,38,39,40]). The numerical simulation results from Molchanov et al. (1995) [41] suggested that the vertical amplitude of the primary magnetic signal from the Earth’s crust is greater than or close to the horizontal amplitude; that is, the ratio of the vertical amplitude to the horizontal amplitude of the magnetic field from the Earth’s crust is greater than or close to 1. Therefore, the abnormal signal from the source area can be highlighted, and the signal from the external field can be suppressed by using the vertical polarization of the geomagnetic intensity.

The physical meaning of the method of identifying anomalies in the seismic direct current apparent resistivity is intuitive, and the mechanism of the stress–strain process is clearly related to earthquake preparation. The observed data have relatively stable background changes, and the seismic anomalies related to earthquake preparation and occurrence can be obtained without using signal processing methods or other complex mathematical operations. For direct current apparent resistivity interference analysis, the potential interference source is generally identified through anomaly field verification, and a physical model is established in combination with the actual electrical structure of the station for qualitative analysis and quantitative calculation to determine the cause of abnormal changes in the original observation data and then eliminate interference changes.

The search for earthquakes or volcanic precursors is based on apparent resistivity monitoring by the relatively high-frequency magnetotelluric method in seismically active areas [35,42]. However, due to the decreasing signal-to-noise level in the electric field with increasing periods, the magnetotelluric method is not reliable enough for long periods on land, and geomagnetic depth sounding methods are better at investigating apparent temporal resistivity changes, especially for low frequencies [35]. The process of preparing for an earthquake is accompanied by changes in rock conductivity, and the strength of the geomagnetic field’s internal field is related to both the strength of the external field and the electrical conductivity of underground materials [43]. Therefore, the change in the electrical conductivity of underground materials caused by stress accumulation will cause a change in the strength of the geomagnetic internal field, that is, a change in the ratio of the external field to the internal field [44]. The variation in the internal and external field ratio can be reflected by the harmonic amplitude ratio of the geomagnetic diurnal variation (e.g., [45]).

In this study, based on the actual demand for short-term earthquake forecasts in the Gansu–Qinghai–Sichuan area, we further refined the construction of earthquake forecast indicators (using the above methods) in view of the abundant electromagnetic anomalies before moderate and strong earthquakes in this area. Combined with significant earthquakes that have occurred in recent years in China, we summarized the advantageous forecast indicators of each method for the three earthquake elements (time, epicenter, and magnitude) and determined the spatiotemporal evolution process of the anomalies. The advantages of each method in spatiotemporal strong parameter forecasting are extracted so that the urgency of subsequent moderate strong earthquakes in this region can be quantified. It is expected that the research results can be used to propose a scientific research and judgment strategy based on integrated earthquake forecasting, especially for short-term forecasts of impending earthquakes in China.

## 3. Results

Based on seismic electromagnetic analysis before 14 M ≥ 5.5 earthquakes occurred in China, we have sorted the electromagnetic precursor anomalies in the Gansu–Qinghai–Sichuan area since 2015, and the results included a total of 90 groups of electromagnetic precursor anomalies. The statistical results show that there are abundant pre-earthquake anomalies preceding M ≥ 5.5 earthquakes, including the 2022 M6.0 Maerkang earthquake, 2022 M6.0 Delingha earthquake, 2022 M6.9 Menyuan earthquake, 2021 M5.5 Akesai 5.5 earthquake, 2021 M7.4 Maduo earthquake, 2019 M5.7 Xiahe earthquake, 2018 M7.0 Jiuzhaigou earthquake, 2016 M6.4 Menyuan earthquake, and 2013 M6.6 Min-Zhang earthquake. Most of the anomalies exhibit continuous time spans and are mainly concentrated near the epicenter. Among these earthquakes, the most significant pre-earthquake anomalies were associated with the 2022 M6.9 Menyuan earthquake and the 2021 M7.4 Maduo earthquake.

### 3.1. 2022 M6.9 Menyuan Earthquake

**Figure 1:**
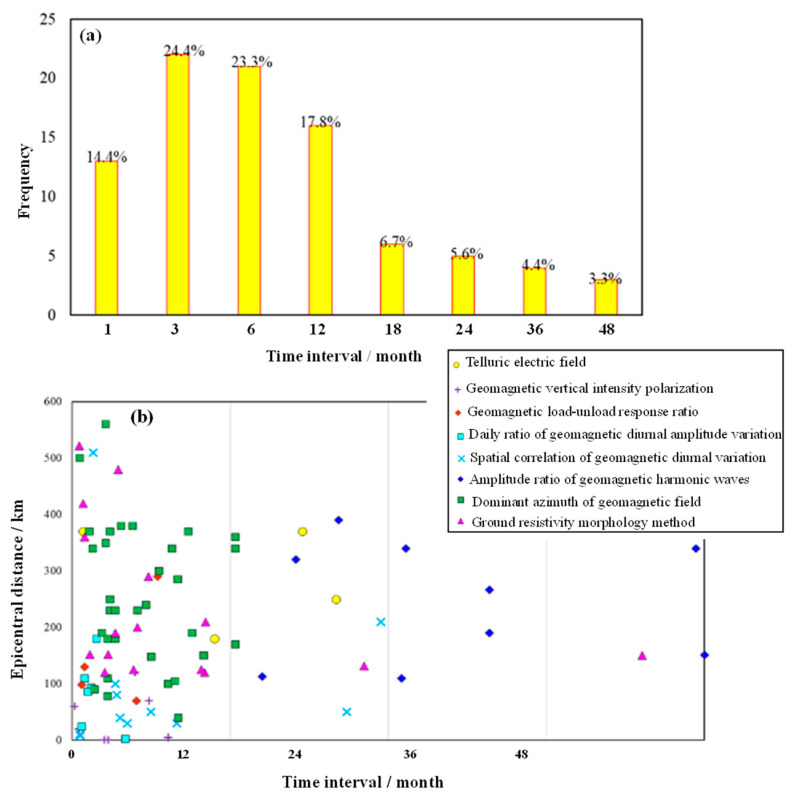
Statistical characteristics of electromagnetic anomalies before M ≥ 5.5 earthquakes in the Gansu–Qinghai–Sichuan area since 2015. (**a**) Relationship between the frequency of earthquakes and the time interval between the occurrence of the electromagnetic anomaly and the occurrence of the earthquake. (**b**) The relationship between the epicentral distance and the time interval between the occurrence of the electromagnetic anomaly and the earthquake.

Before the occurrence of the M6.9 Menyuan earthquake on 8 January 2022, a total of eight electromagnetic anomalies were detected in Qinghai and neighboring areas (Figure 1, e.g., [24]), including spatial correlations between the Dulan geomagnetic field, the Menyuan geomagnetic field dominant azimuth, the Baishui River geomagnetic field dominant azimuth, the Dawu geomagnetic field dominant azimuth, the geomagnetic vertical intensity polarization on 27 October 2022, the apparent resistivity of the Shandan magnetic sounding, the daily ratio of the geomagnetic field diurnal variation on 25 November 2021, and the geomagnetic diurnal variation on 22 April 2020. We concluded that the spatiotemporal evolution characteristics of the above six anomalies are highly correlated.

The earliest anomaly was the spatial correlation of geomagnetic diurnal variation on 22 April 2020, which was followed by the apparent resistivity of the Dulan field and Shandan magnetic sounding, and the dominant azimuth of the Menyuan field and Dawu field appeared 3 to 5 months before the occurrence of the earthquake. Three months before the occurrence of the earthquake, the vertical intensity polarization of the geomagnetic field on 27 October 2022, the dominant azimuth of the geomagnetic field in the Baishui River on 25 November 2021, and the daily ratio of the geomagnetic field amplitude on 25 November 2021, successively exhibited anomalies. In terms of spatial distribution, the epicentral distance of each method from near to far was approximately 20 km for vertical geomagnetic intensity polarization, approximately 40 km for the dominant azimuth of the Menyuan geomagnetic field, 50 km for the spatial correlation of geomagnetic diurnal variation on 22 April 2020, 93 km for the daily ratio of geomagnetic diurnal variation on 25 November 2021, and 113 km for the apparent resistivity of Shandan magnetic sounds. The dominant azimuth of the Dulan electric field and the Baishui River electric field is approximately 370 km, and the dominant azimuth of the Dawu electric field is approximately 380 km.

### 3.2. 2021 M7.4 Maduo Earthquake

Before the occurrence of the 2021 Maduo M7.4 earthquake, abundant electromagnetic precursor anomalies were extracted in Qinghai (Figure 1, e.g., [22]), including anomalies in the vertical intensity polarization on 15 October 2020, and 7 May 2021; anomalies in the response ratio of geomagnetic loading and unloading on 26 December 2020; spatial correlation anomalies in the diurnal variation in the geomagnetic field from 26 to 28 September 2020; anomalies in the dominant azimuth of the Dawu electric field; and anomalies in the Huangyuan geomagnetic harmonic amplitude ratio. We analyzed the temporal and spatial evolution characteristics of the above six anomalies one by one. The earliest anomaly was the Huangyuan harmonic amplitude ratio anomaly, which occurred approximately 3.8 years before the occurrence of the earthquake, followed by the Dawu electric field dominant azimuth anomaly, which occurred approximately one year before the occurrence of the earthquake. From September to December 2020, spatial correlation anomalies in the geomagnetic diurnal variation, geomagnetic vertical intensity polarization anomalies, and geomagnetic loading and unloading response ratio anomalies successively appeared, and short-term anomalies in the geomagnetic vertical intensity polarization reappeared 15 days before the occurrence of the earthquake. From the perspective of spatial distribution, the six anomalies were concentrated near the epicenter of Madao, and the epicentral distance of each index from near to far was approximately 20 km for geomagnetic vertical intensity polarization, 30 km for geomagnetic daily variation, 70 km for the geomagnetic loading and unloading response ratio, 175 km for the dominant azimuth angle of the Dawu electric field, and approximately 340 km for the geomagnetic harmonic amplitude ratio of Huangyuan.

### 3.3. Statistics of Electromagnetic Anomalies before 14 M ≥ 5.5 Earthquakes

We have surveyed the temporal and spatial characteristics of electromagnetic anomalies before 14 M ≥ 5.5 earthquakes in the Gansu–Qinghai–Sichuan region since 2015. In general, the electromagnetic anomalies appeared intensively and synchronously before the occurrence of an earthquake and gradually disappeared after the occurrence of the earthquake, and the anomalous areas were concentrated near the epicenter. Approximately 80% of the electromagnetic anomalies occurred within a year before the earthquake, and 62% of the electromagnetic anomalies occurred within six months before the earthquake. In addition, from the perspective of the spatial distance of the earthquake (Figure 1, e.g., [28,46]), electromagnetic anomalies mainly occurred within 400 km of the associated earthquake, and 77% of the electromagnetic anomalies occurred within 300 km of the earthquake. Therefore, the electromagnetic anomalies in the Gansu–Qinghai–Sichuan area mainly occurred within 300 km of the earthquake epicenter and within half a year before the occurrence of the earthquake.

As shown in Table 1, we surveyed the accuracy of the forecast indicators for earthquakes in China and the Gansu–Qinghai–Sichuan area. The five methods (marked with “#”) show advantages in terms of the accuracy of earthquake occurrence time and epicenter forecasts and act as good indices for the Gansu–Qinghai–Sichuan area.

**Figure 2:**
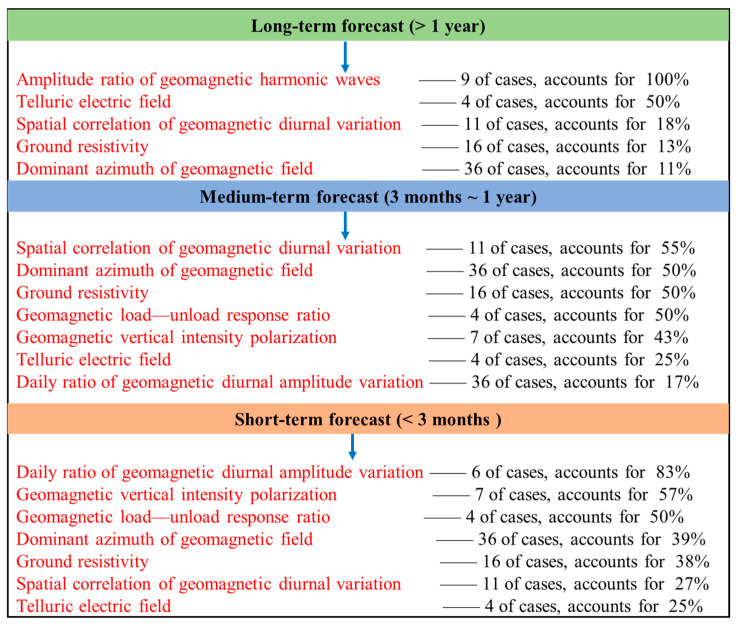
Accuracy of electromagnetic forecast indicators for predicting earthquakes within a given timeframe.

**Figure 3:**
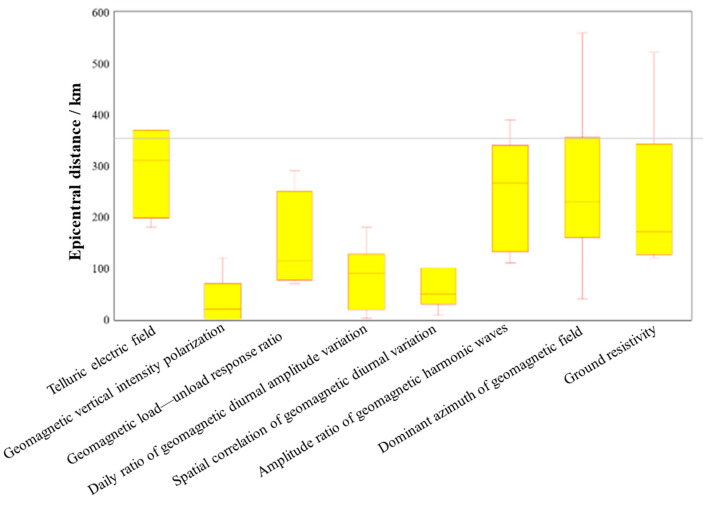
Electromagnetic forecast indicator accuracy in predicting earthquake epicenters. <100 km: geomagnetic vertical intensity polarization, daily ratio of geomagnetic diurnal amplitude variation, spatial correlation of geomagnetic diurnal variation; 100~350 km: telluric electric field, geomagnetic load–unload response ratio, amplitude ratio of geomagnetic harmonic waves, dominant azimuth of geomagnetic field, ground resistivity; >350 km: amplitude ratio of geomagnetic harmonic waves, dominant azimuth of geomagnetic field, ground resistivity.

According to the long-term, medium-term, and short-term forecasts, we calculated probability statistics for all earthquake-related methods (Figure 2) and obtained the accuracy rate of each method during each forecasting period. The probabilities of the three methods in short-term forecasting reached more than 50%, indicating relatively good short-term forecasting. In addition, in terms of spatial distribution (Figure 3), there are three methods with better alignment rates within the range of 100 km from the anomaly, namely geomagnetic vertical intensity polarization, the daily ratio of geomagnetic diurnal amplitude, and the spatial correlation of geomagnetic diurnal variation.

**Figure 4:**
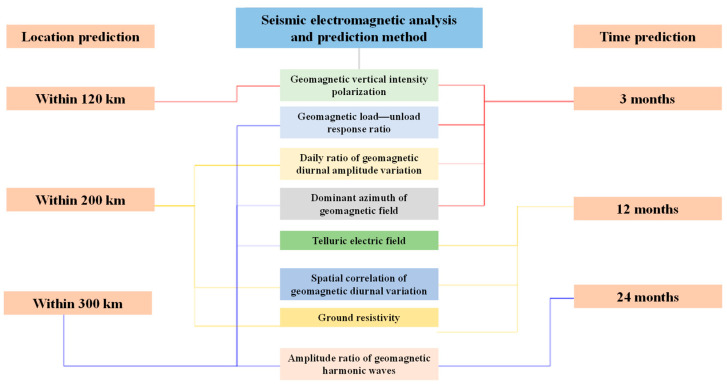
Comprehensive forecast index evaluation via this study.

Based on the above results, the advantages of the eight electromagnetic analysis and forecast methods are classified according to their spatial and temporal forecasting abilities (Figure 4). In terms of temporal forecasting, the geomagnetic vertical intensity polarization method, geomagnetic load–unload response ratio method, geomagnetic daily ratio method, and geomagnetic field dominant azimuth method are the most effective at predicting M ≥ 5.5 earthquakes in the Gansu–Qinghai–Sichuan areas within 3 months and are short-term forecasting methods. The electromagnetic anomalies detected by the telluric electric field method, geomagnetic diurnal spatial correlation method, and geomagnetic resistivity pattern method mainly appeared approximately 12 months before the occurrence of the earthquake. The geomagnetic harmonic amplitude ratio is used as a background anomaly tracking method for annual-scale forecasts.

In terms of the epicenter forecast, the geomagnetic vertical intensity polarization method mainly indicates the occurrence of M ≥ 5.5 earthquakes within 120 km of the threshold line, while the daily ratio of geomagnetic diurnal variation, the spatial correlation of geomagnetic diurnal variation, and the geomagnetic resistivity morphological method are better for the forecast of moderate and strong earthquakes within 200 km of the forecast area. The response ratio of geomagnetic loading–unloading and the amplitude ratio of geomagnetic harmonic waves mainly indicate the occurrence of M ≥ 5.5 earthquakes within 300 km of the forecast area. It is expected that these results will play a positive role in the next step of research on short-term to imminent earthquake conditions in the Gansu–Qinghai–Sichuan area, especially in the Qinghai area.

Based on the above analysis, we found that the combination of five methods, including the daily ratio of geomagnetic diurnal variation amplitude, geomagnetic vertical intensity polarization, geomagnetic loading and unloading response ratio, geomagnetic diurnal variation spatial correlation, and geomagnetic field dominant azimuths, has great advantages in short-term earthquake forecasting, with good accuracy in terms of occurrence time and epicenter location.

## 4. Discussion

The problem of earthquake forecasting consists of consecutive, step-by-step narrowing of the time interval, location, and magnitude, and finding a reliable seismic precursor is one of the major challenges for the global scientific community [47,48]. Earthquake forecasts can be classified into three categories: long-term (10 to 100 years), intermediate-term (1 to 10 years), and short-term (e.g., [10]). Long-term forecasts involving a timescale of hundreds to thousand years can be studied based on plate tectonics, earthquake activity, and fault records, and medium-term forecasts involving a few decades to a few years can be achieved by utilizing seismicity and crustal movement databases (e.g., [49]). However, short-term earthquake forecasts based on reliable earthquake precursors are highly valuable, although short-term earthquake forecasts are more difficult than long-term and intermediate-term forecasts [50]. There were very few electromagnetic precursor findings available before 1995, and the electromagnetic radiation in the low-frequency band [9] and direct current seismic electric signals [51] were relatively high [52]. Studies carried out in recent decades have led to the new field of seismo-electromagnetism showing evidence of electromagnetic emissions and anomalies before earthquakes, and researchers have narrowed their studies related to electromagnetic effects from long-term forecasts to short-term forecasts (e.g., [53]). In terms of electromagnetic pre-earthquake precursors, there have been many positive reports on electromagnetic phenomena at a wide frequency range in many parts of the world, including Greece, Japan, Russia, China, Armenia, Italy, and Mexico (e.g., [54,55,56,57]). These aspects of research indicate the significance of electromagnetic effects on short-term earthquake precursors, and short-term earthquake precursors related to electromagnetic effects may be considered one of the highest priorities for social demands in forecast-based earthquake disaster risk reduction strategies in earthquake-prone countries (e.g., [48,49,57,58]).

Due to the limited number of earthquake cases, the understanding of the indicators may be insufficient, but with the accumulation of observation data and earthquake cases, the existing forecast indicators will be further improved, and they are expected to provide some reference value in actual earthquake forecast work in the future. It is necessary to pay close attention to the variations in electromagnetic observations in a region, especially short-term anomalies, to provide a powerful criterion for the forecast of these three earthquake elements. It is necessary to pay attention to the commonality and characteristics of different anomalies and summarize long-, medium-, and short-term earthquake forecast methods to improve the timeliness of regional earthquake forecasts.

## Figures and Tables

**Table 1:** Statistics of the accuracy of electromagnetic methods for predicting earthquakes.

	Method	Telluric Electric Field	Daily Ratio of Geomagnetic Diurnal Amplitude Variation ^#^	Geomagnetic Vertical Intensity Polarization ^#^	Geomagnetic Load–Unload Response Ratio ^#^	Spatial Correlation of Geomagnetic Diurnal Variation ^#^	Amplitude Ratio of Geomagnetic Harmonic Waves	Dominant Azimuth of Geomagnetic Field ^#^	Ground Resistivity Morphology Method
Element									
National forecasting index	Eq time *	none	6~9 m	6~12 m	6~9 m	21 m	18 m	3~6 m	60 d (M ≥ 5) 160 d (M ≥ 6, <150 km) 400 d (M ≥ 7, <200 km) 2 yr (M ≥ 8, <300 km)
	Epicenterdistance	none	300 km	High-value region	300 km	300 km	250 km	300 km	
	Mag	none	M ≥ 6 (WC) M ≥ 5 (EC)	DoAA	M ≥ 6 (WC) M ≥ 5 (EC)	M ≥ 6 (WC) M ≥ 5 (EC)	M ≥ 6 (WC) M ≥ 5 (EC)	M ≥ 4.5 (WC) M ≥ 4 (EC)	
Qinghai–Gansu–Sichuan forecasting index	Eq time *	12 m	3 m	3 m	3 m	12 m	24 m	3 m	12 m
	Epicenter distance	300 km	200 km	120 km	300 km	200 km	300 km	300 km	200 km
	Mag	M ≥ 5.5	M ≥ 6	DoAA	M ≥ 6	M ≥ 6	M ≥ 6	M ≥ 5.5	M ≥ 5.5

^#^: Five methods showing advantages in terms of the accuracy of earthquake occurrence time and epicenter forecasts in the Gansu–Qinghai–Sichuan area. *: Eq time indicates the average time between the occurrence of anomalies and the occurrence of M ≥ 5.5 earthquakes. Abbreviations: WC: western China; EC: eastern China; DoAA: depend on the area of anomalies.

## Data Availability

Data are available in the figures and tables of this paper.

## References

[1] Tiampo K.F., Shcherbakov R. (2012). Seismicity-based earthquake forecasting techniques: Ten years of progress. Tectonophysics.

[2] Jordan T.H. (2006). Earthquake predictability, brick by brick. Seismol. Res. Lett..

[3] Davis C., Keilis-Borok V., Kossobokov V., Soloviev A. (2012). Advance forecast of the March 11, 2011 Great East Japan Earthquake: A missed opportunity for disaster preparedness. Int. J. Disaster Risk Reduct..

[4] Turcotte D.L. (1991). Earthquake forecast. Annual Review of Earth and Planetary Sciences.

[5] Scholz C.H. (2019). The Mechanics of Earthquakes and Faulting.

[6] Chen H., Han P., Hattori K. (2022). Recent advances and challenges in the seismo-electromagnetic study: A brief review. Remote Sens..

[7] Huang Q. (2002). One possible generation mechanism of coseismic electric signals. Proc. Jpn. Acad. Ser. B.

[8] Ren H., Chen X., Huang Q. (2012). Numerical simulation of coseismic electromagnetic fields associated with seismic waves due to finite faulting in porous media. Geophys. J. Int..

[9] Gokhberg M.B., Morgounov V.A., Yoshino T., Tomizawa I. (1982). Experimental measurement of electromagnetic emissions possibly related to earthquakes in Japan. J. Geophys. Res. Solid Earth.

[10] Hayakawa M., Hobara Y. (2010). Current status of seismo-electromagnetics for short-term earthquake prediction. Geomat. Nat. Hazards Risk.

[11] Hayakawa M., Schekotov A., Izutsu J., Nickolaenko A.P. (2019). Seismogenic effects in ULF/ELF/VLF electromagnetic waves. Int. J. Electron. Appl. Res..

[12] Sharma S., Swati Singh R.P., Pundhir D., Singh B. (2021). Lithospheric electromagnetic emissions associated with some major earthquakes occurred in indian subcontinent. Geomagn. Aeron..

[13] Hayakawa M., Hattori K. (2004). Ultra-low-frequency electromagnetic emissions associated with earthquakes. IEEJ Trans. Fundam. Mater..

[14] Zhang P.T., Feng L.L., Li X., Zhao Y.H., Liu L. (2022). Relationship between lithospheric magnetic field characteristics and strong earthquakes in the Qinghai area. Seismol. Geomagn. Obs. Res..

[15] Yuan G.P., Zhang X.M., Wu Y.Y., Zhao X.D. (2015). Minimum point shift of the geomagnetic vertical component in diurnal variation and the internal-external equivalent current system Sq before the 2008 Wenchuan Ms8. 0 Earthquake. Earthquake.

[16] Xie T., Liu J., Lu J., Li M., Yao L., Wang Y., Yu C. (2018). Retrospective analysis on electromagnetic anomalies observed by ground fixed station before the 2008 Wenchuan M S 8.0 earthquake. Chin. J. Geophys..

[17] Zhao Y.H., Su W.G., Feng L.L., Li X., Liu L., Sun X.H. (2023). Characteristics of the dominant azimuth anomalies of geoelectric field at Dawu station before M S 7.4 Maduo, Qinghai earthquake in 2021. Acta Seismol. Sin..

[18] Xie T., Yu C., Wang Y.L., Li M., Wang Z.P., Yao L., Lu J. (2022). Apparent resistivity variation of tongwei seismic station before the minxian-zhangxian Ms6.6 earthquake in 2013. Seismol. Geol..

[19] Liao X., Fan W., Qiu G., Li X., Yang P. (2021). Analysis on Short-Term Characteristics of Geomagnetic Vertical Intensity Polarization Anomaly before the Jiuzhaigou 7.0 Earthquake on August 8, 2017. Earthquake.

[20] Li X., Feng L.L., Zhao Y.H., Liu L., Gou Z.D., Fan W.J., He M.Q., Liao X.F., Aisa Y. (2021). Anomalous characteristics of geomagnetic vertical strength polarization before the 2017 Milin Ms 6.9 earthquake in Tibet. Acta Seismol. Sin..

[21] Ni X.Y., Huang S., Jiang C.F. (2021). Geomagnetic Diurnal Variation Anomalies Before 2019 Changning M6.0 Earthquake and Xiahe M5.7 Earthquake. Earthquake.

[22] Li X., Feng L., Zhao Y., Liu L., Gou Z., Dai M. (2022). Analysis on characteristics of geomagnetic load-unload response ratio method before Maduo Ms7.4 earthquake. Prog. Geophys..

[23] Xin J.C., Sun J.S., Yu C., Fang W., Zhao J., Yang Y.H. (2022). Abnormal variation of dominant azimuth and load/unload response ratio of geoelectric field before the Menyuan MS6.9 earthquake. China Earthq. Eng. J..

[24] Fan W.J., Feng L.L., Li X., He C., Liao X.F., Yao X.Y. (2022). Characteristics of geomagnetic vertical intensity polarization anomalies before Menyuan, Qinghai Ms6. 9 earthquake on 8 January 2022. J. Earthq. Eng. J.

[25] Xu G., Han P., Huang Q., Hattori K., Febriani F., Yamaguchi H. (2013). Anomalous behaviors of geomagnetic diurnal variations prior to the 2011 off the Pacific coast of Tohoku earthquake (Mw9. 0). J. Asian Earth Sci..

[26] Ding J.H., Liu J., Yu S.R., Xiao W.J. (2004). Geomagnetic diurnal-variation anomalies and their relation to strong earthquakes. Acta Seismol. Sin..

[27] Han P., Hattori K., Huang Q., Hirooka S., Yoshino C. (2016). Spatiotemporal characteristics of the geomagnetic diurnal variation anomalies prior to the 2011 Tohoku earthquake (Mw 9.0) and the possible coupling of multiple preearthquake phenomena. J. Asian Earth Sci..

[28] Feng L., Qu R., Ji Y., Zhu W., Zhu Y., Feng Z., Fan W., Guan Y., Xie C. (2022). Multistationary geomagnetic vertical intensity polarization anomalies for predicting M ≥ 6 earthquakes in Qinghai, China. Appl. Sci..

[29] Yang C.H., Cheng P.H., You J.I., Tsai L.L. (2002). Significant resistivity changes in the fault zone associated with the 1999 Chi-Chi earthquake, west-central Taiwan. Tectonophysics.

[30] Du X. (2011). Two types of changes in apparent resistivity in earthquake forecast. Sci. China Earth Sci..

[31] Du X.B., Liu J., Cui T.F., Fan Y.Y., An Z.H., Yan R., Wang L. (2015). Repeatability, similarity and anisotropy changes in apparent resistivity recorded by station Chengdu at near distances before two great earthquakes. Chin. J. Geophys..

[32] Sapia V., Villani F., Fischanger F., Lupi M., Baccheschi P., Pantosti D., Pucci S., Civico R., Sciarra A., Smedile A. (2021). 3-d deep electrical resistivity tomography of the major basin related to the 2016 mw 6.5 central Italy earthquake fault. Tectonics.

[33] Zhang J., Wu X., Yang X., Du W., Yue M. (2017). Observational evidence of anisotropic changes of apparent resistivity before strong earthquakes. Geophys. J. Int..

[34] Lilley F.E.M., Tammemagi H.Y. (1972). Magnetotelluric and geomagnetic depth sounding methods compared. Nat. Phys. Sci..

[35] Petrishchev M.S., Semenov V.Y. (2013). Secular variations of the Earth’s apparent resistivity. Earth Planet. Sci. Lett..

[36] Baker D.N., Klimas A.J., McPherron R.L., Büchner J. (1990). The evolution from weak to strong geomagnetic activity: An interpretation in terms of deterministic chaos. Geophys. Res. Lett..

[37] Li Q., Schekotov A., Asano T., Hayakawa M. (2015). On the anomalies in ULF magnetic field variations prior to the 2008 Sichuan earthquake. Open J. Earthq. Res..

[38] He C., Feng Z. (2017). Application of polarization method to geomagnetic data from the station Chengdu. Acta Seismol. Sin..

[39] Zhang M., Zhao S., Jia L., Liu L., Teng Y. (2020). Applying of Polarization Method to Extract Short Term Seismic Anomaly from Geomagnetic Second Data. Prog. Geophys..

[40] Zhang L., Chen C., Gong J., Guo Y., Fan L., Shi S. (2023). Anomaly characteristics of Shanxi geomagnetic array data before Yuanping M4.2 earthquake. Prog. Earthq. Sci..

[41] Molchanov O.A., Hayakawa M. (1995). Generation of ULF electromagnetic emissions by microfracturing. Geophys. Res. Lett..

[42] Lu J., Qian F., Zhao Y. (1999). Sensitivity analysis of the Schlumberger monitoring array: Application to changes of resistivity prior to the 1976 earthquake in Tangshan, China. Tectonophysics.

[43] Freund F. (2011). Preearthquake signals: Underlying physical processes. J. Asian Earth Sci..

[44] Li X., Feng L.L., Zhao Y.H., Gou Z.D. (2022). Anomaly Indicators Extracted of Geomagnetism Harmonic Amplitude Ratio Based on Rate Accumulation Algorithm. J. Geod. Geodyn..

[45] Lu Q.Q., Ding J.H. (1998). The Effect of Lunar Phase on Aftershock Activity of Taiwan Strait Earthquake with M~s7.3. Earthquake.

[46] Faheem H., Li X., Zhu W., Ji Y., Feng L., Zhu Y. (2024). Refinement of different frequency bands of geomagnetic vertical intensity polarization anomalies before m> 5.5 earthquakes. Sensors.

[47] Keilis-Borok V., Soloviev A.A. (2002). Nonlinear Dynamics of the Lithosphere and Earthquake Forecast.

[48] Petraki E., Nikolopoulos D., Nomicos C., Stonham J., Cantzos D., Yannakopoulos P., Kottou S. (2015). Electromagnetic preearthquake precursors: Mechanisms, data and models-A review. J. Earth Sci. Clim. Chang..

[49] Hayakawa M. (2018). Earthquake precursor studies in Japan. Pre-Earthquake Processes: A Multidisciplinary Approach to Earthquake Forecast Studies.

[50] Peresan A., Kossobokov V., Romashkova L., Panza G.F. (2005). Intermediate-term middle-range earthquake forecasts in Italy: A review. Earth-Sci. Rev..

[51] Varotsos P., Alexopoulos K. (1984). Physical properties of the variations of the electric field of the earth preceding earthquakes, I. Tectonophysics.

[52] Hayakawa M. (2019). Seismo electromagnetics and earthquake forecast: History and new directions. Int. J. Electron. Appl. Res..

[53] Khan P.A., Tripathi S.C., Mansoori A.A., Bhawre P., Purohit P.K., Gwal A.K. (2011). Scientific efforts in the direction of successful Earthquake Forecast. Int. J. Geomat. Geosci..

[54] Balassanian S., Mouradian A., Sahakian A., Kalinin S., Babayan M., Pogossian A. (1997). The Investigation of Electromagnetic Precursors to Earthquakes in Armenia. Ann. Geophys..

[55] Hattori K. (2004). ULF geomagnetic changes associated with large earthquakes. Terr. Atmos. Ocean. Sci..

[56] Kamogawa M. (2006). Preseismic lithosphere-atmosphere-ionosphere coupling. Eos Trans. Am. Geophys. Union.

[57] Uyeda S., Nagao T., Kamogawa M. (2009). Short-term earthquake prediction: Current status of seismo-electromagnetics. Tectonophysics.

[58] Conti L., Picozza P., Sotgiu A. (2021). A critical review of ground based observations of earthquake precursors. Front. Earth Sci..

